# Landscape changes and livelihood outcomes in rural tea farming communities: A case study in Fuding City, Fujian Province, Southeast China

**DOI:** 10.1371/journal.pone.0295620

**Published:** 2023-12-12

**Authors:** Chengchao Wang, Xianqiang Song, Dongshen Luo, Xu Dan, Tingting Lin

**Affiliations:** 1 Foshan University, Foshan, P. R. China; 2 Key Laboratory for Humid Subtropical Eco-Geographical Processes of the Ministry of Education, Fujian Normal University, Fuzhou, P. R. China; Chulalongkorn University, THAILAND

## Abstract

Landscape changes driven by cash crop plantations have been prevalent in tropical and subtropical regions worldwide in recent decades. Investigating the landscape changes and concluding livelihood outcomes are fundamental to figure out the solutions for rural sustainability. This paper examined the landscape changes which was caused by land use changes in tea plantations as well as investigated the resultant livelihood impacts, based on a case study in Fuding City, Southeast China. A questionnaire survey of 114 rural households in four sampled villages was conducted. Results demonstrated that expansion and intensification of tea plantations were two major proximate causes of landscape changes in recent decade. Our survey indicated that some existing intensively-managed tea plantations had derived from intensification and expansion of tea plantations, respectively. We identified four underlying driving forces of landscape changes, including economic benefit, governmental policies, wildlife destruction on grain crops, and rural return migration. Our study confirmed that landscape changes have significant positive effects on farmers’ livelihoods, including increasing employment and incomes, raising living standards, enhancing livelihood assets and livelihood sustainability. Especially, the aged rural populations could have a relatively decent living standard. Meanwhile, the excessive expansion of tea plantations may impair livelihood resilience. Lastly, three policy suggestions based on different time scales have been put forward to promote rural households’ livelihood sustainability and resilience.

## 1. Introduction

The global countryside has undergone significant landscape changes over the past decades. Two converse trends of land use changes—intensification and extensification, together with land abandonment have remarkably promoted the process of landscape transition [1, 2]. Cash crop expansion has recently been a major land use change in tropical and subtropical regions worldwide. The agricultural transition from planting traditional grain crops to market-oriented cash crops, such as rubber, oil palm, coffee, tea, and other commodities, has substantially transformed rural landscapes in tropical and subtropical developing countries since the later 20^th^ century [3–6]. It has been widely reported that substantial natural forest has turned into cash crop plantations in many developing countries of Southeast Asia, South Asia and East Asia [7, 8]. The process of land use change has brought about profound environmental and socioeconomic impacts on reduction in water conservation, biodiversity, soil productivity, carbon stocks, and food security, livelihood resilience of small-holders [9–13]. Livelihood resilience is defined as the capacity of all people across generations to sustain and improve their livelihood opportunities and well-being despite environmental, economic, social and political disturbances [14]. Given the significance of cash crop expansion and spatial heterogeneity, it is necessary to carry out more local studies.

Existing research demonstrated that multi-scale factors stimulate the expansion of cash crops. Firstly, terrain, elevation, slope, soil fertility and accessibility are primary driving forces of cash crop expansion at the parcel scale [15, 16]. Secondly, high returns, labour availability, economic situations, land quantity, knowledge and skills are regarded as principal drivers at household scale [4, 7]. Among these driving factors, economic profit of cash crop plantations is the decisive one. Thirdly, resource endowments, accessibility, location (especially proximity to towns and cities), and village characteristics are main stimulation of cash crop expansion at village scale [5, 7]. Fourthly, globalization, population growth, development of macro economy, decreasing costs of transportation and communications, and governmental policies have been playing a great role in cash crop expansion at macro scale [4, 17, 18]. In fact, cash crop expansion has been primarily conducted by numerous farmers, but studies researched the primary drivers of cash crop expansion from the perspective of farmers are few.

Landscape Changes driven by cash crop expansion have brought about remarkable livelihood impacts on local rural households. A livelihood comprises the capabilities, assets (stores, resources, claims and access) and activities required for a means of living. A livelihood is sustainable which can cope with and recover from stress and shocks, maintain or enhance its capabilities and assets and provide sustainable livelihood opportunities for the next generation [19]. Cash crop expansion has been claimed not only to affect rural current livelihoods but also livelihood sustainability [20]. Proponents considered that high profits of cash crop plantations could alleviate poverty and revitalize rural economy [4, 21–23]. Opponents argued that livelihood resilience of smallholders could be negatively affected owing to simplified livelihoods, narrowing of income sources, price fluctuations of cash crops, potential loss of food security, macro-economic fluctuations and dependency on uncertain global markets [13, 20, 24]. The research disputes attributed to a series of causes, such as spatial heterogeneity, price fluctuations of cash crop products, temporal difference, crop diversity, farm scale, etc. Spatial heterogeneity can be defined generally as the complexity and variability of a system property in space. While a system property can be anything, such as vegetation, plant biomass, or soil nutrients. Thus, spatial heterogeneity is a universal phenomenon, existing in ecological systems and human-environment system at all scales [25]. Rural livelihoods in areas with high extent of cash crop expansion may be positively affected in terms of increasing employment and incomes in short period. But the livelihood resilience may be negatively affected. Moreover, the livelihood impacts of cash crop expansion are not only indicated in overall employment and incomes, but also in poverty alleviation, improvement of livelihood assets and livelihood sustainability [26]. However, there are few studies conducted comprehensive appraisal of livelihood impacts.

Cash crop plantations have been rapidly expanding in China during recent decades. Investigations of three Chinese National Land Survey showed that the acreage of cash crop plantations increased from 10,023.8 thousand hectares in the end of 1996, to 14,812.0 thousand hectares in the end of 2009, increasing by 47.77% in 13 years. The Third Chinese National Land Survey revealed that the area of cash crop plantations was 20,171.6 thousand hectares in the end of 2019, increasing by 36.18% in 11 years. Among them, the acreage of tea plantations was 13,031.3 thousand hectares, which accounted for 8.35% of the total in 2019. Tea plantations have experienced rapid expansion during the past decades. The area of tea plantations increased from 169 thousand hectares in 1950 [17] to 13,031.3 thousand hectares in 2019, increasing by 8.97 times during past seven decades. The tea plantations are substantially distributed in subtropical areas of China, such as Fujian, Zhejiang, Guizhou, and Yunnan Province. The economic benefits of tea plantation expansion have been discussed in previous studies [4, 27]. However, less study systematically illustrated the livelihood impacts of tea plantation expansion, especially for the disadvantaged groups, such as the aged and rural women.

Considering that cash crop plantations is the fundamental source of income for the peasants, but the researches that can clarify the change of landscape and whether the landscape change would improve the livelihoods of the peasants are limited, this paper attempted to investigate the landscape changes and resultant livelihood impacts based on a case study in Fuding City, Fujian Province, Southeast China. Our primary objectives in this study are: (1) illustrate the manifestation of landscape changes; (2) clarify the underlying drivers of land use changes; (3) examine livelihood impacts of landscape changes based on the framework of Sustainable Livelihood Approach (SLA), from the perspective rural households. The framework linked inputs (livelihood assets) and outputs (livelihood strategies), connected in turn to livelihood outcomes, which offered an innovative way to think holistically about the objectives, scale, and priorities of development [28, 29]. By virtue of SLA, the impacts of farmers’ livelihoods could be divided into three parts: livelihood assets, livelihood strategies and livelihood outcomes [29].

## 2. Methodology

### 2.1 Conceptual framework

**Fig 1:**
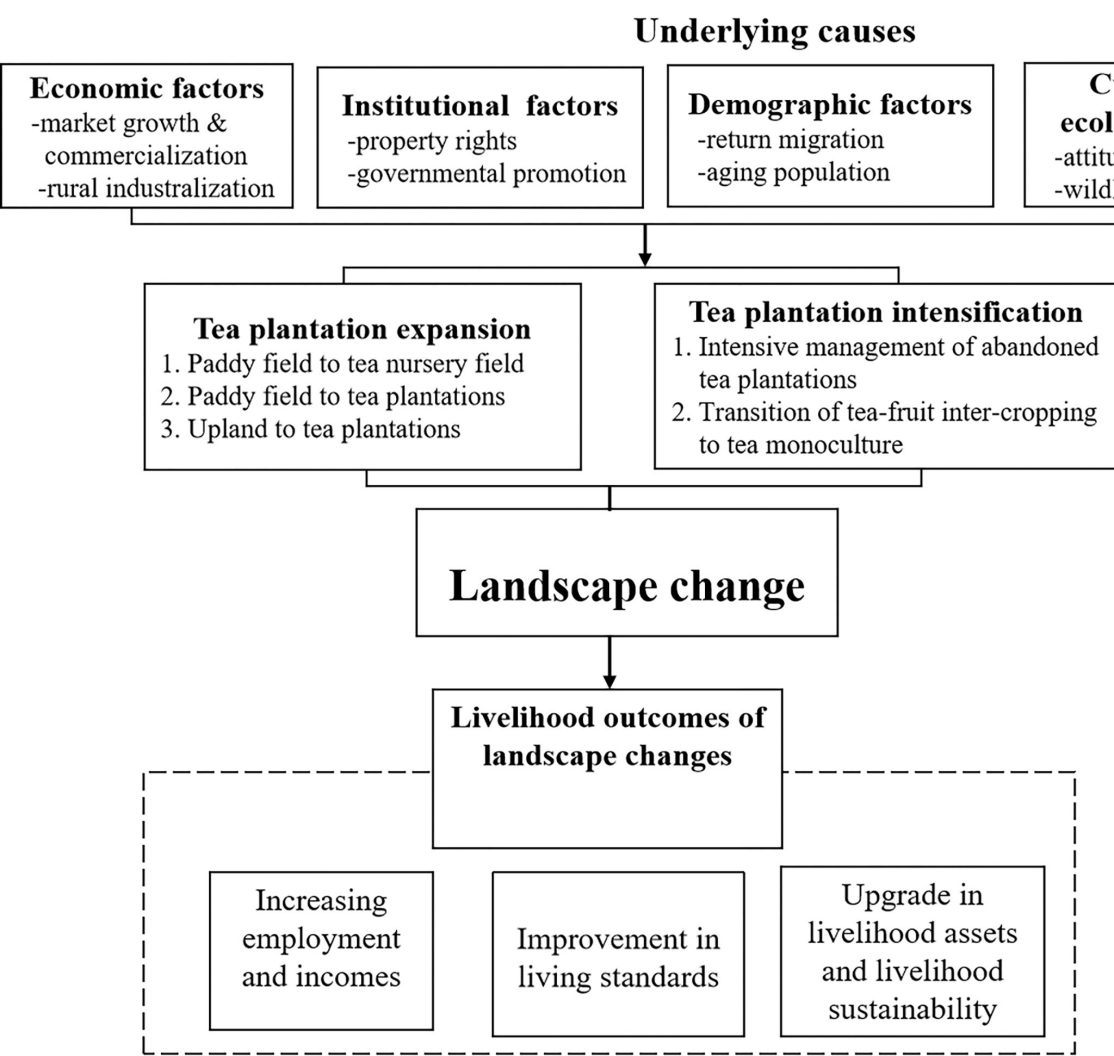
Causes and livelihood outcomes of landscape changes.

Before proceeding to the empirical analysis, we conceptualized the causes and livelihood outcomes of landscape changes using the theoretical framework (Fig 1).

The causes of land use changes in tea plantations are illustrated in terms of proximate and underlying causes by referring to previous studies [30]. While the framework of livelihood outcomes of landscape changes is referred to the Sustainable Livelihoods Framework, and it generally includes income, well-being, and sustainability [19]. Proximate causes of land use changes involving of tea planting were identified: expansion of tea plantation and intensification of tea plantation. Proximate causes are driven by many related underlying causes. Underlying causes were generally categorized into four categories: economic, policy and institutional, demographic, and cultural factors. Each underlying cause could further be subdivided into specific factors which are substantially site-specific. Furthermore, the livelihood outcomes of expansion of cash crops are elucidated in three aspects: employment and incomes, living standards, and livelihood sustainability (Fig 1).

### 2.2 Field survey and data collection

The survey data were collected from October 5, 2021 to November 10, 2021. *Diantou Town* is selected as the typical sampling area because it is the largest town of tea plantations in Fuding City. Then a two-stage random sampling technique was used to select the respondents of the study. In the first stage, four sampling villages were randomly chosen to be our sources. In the second stage, they were selected randomly for investigation after an inventory of households was enumerated. As a result, there are 120 rural households were selected as our interviewees. Meanwhile, two village leaders in each sampling village were selected as our in-depth interviewees. Finally, we chose 8 village leaders and 120 rural households as our field survey’s respondents.

We have obtained official permission from top to bottom for our whole fieldwork. First, the introduction letter of our survey approved by our affiliations has been delivered to the administrating office of Fu’an City to get the survey permission. Second, after finishing the city-level field-surveys, the clerks of administrating office of Fu’an City would convey the permission of our survey to the administrating offices of sampled towns and ask them to cooperate with us during the fieldwork. Third, for the rural respondents, we provided the township government survey permission and explained the purpose of the study and assured them the anonymity, confidentiality and privacy of the responses. All research respondents voluntarily agreed to participate in our study. Consent was obtained from all respondents through verbal agreement.

The microcosmic perspective was highlighted in the study. Survey data were obtained from questionnaire surveys and in-depth interviews were utilized to carry out above studies. Firstly, we conducted a face to face semi-structured interview, which cost about two hours, with each of village leaders. These in-depth interviews mainly focused on the process of tea plantation expansion, driving forces, distribution of tea plantations, smallholder management, and livelihood outcomes. Secondly, the sampled households were interviewed with a questionnaire that included 54 questions and 3 sections: (1) household demographic conditions and consumption situations; (2) land use changes in tea plantation during past two decades, mainly including expansion of tea plantation and tea intensification; (3) livelihood effects of tea expansion and intensification. The questionnaire is designed based on the front conceptual framework: proximate causes, underlying causes, and livelihood outcomes of tea expansion and intensification (Fig 1). For all income or expense variables, the reference period in the questionnaire is the past 12 months. These questionnaires were carried out by our professional investigators through a question-and-answer format to ensure the accuracy of the survey. And we obtained the verbal informed consent from all the interviewees in our study. Each questionnaire survey cost about twenty to thirty minutes. After eliminating 6 invalid records, we gained 114 effective questionnaires. The distribution of investigated households could be seen Table 1. The interview data could provide reference and support for the interpretation of our research results.

**Table 1:** The distribution of respondents in Fuding City, Fujian Province, China.

Sampling village	Town	Survey household	Of total household (%)
Guanyang	Diantou	22	5.70
Wengxi	Diantou	40	10.36
Daping	Diantou	22	4.49
Bailiu	Diantou	30	6.17
Total		114	2.72

### 2.3 Study area

**Fig 2:**
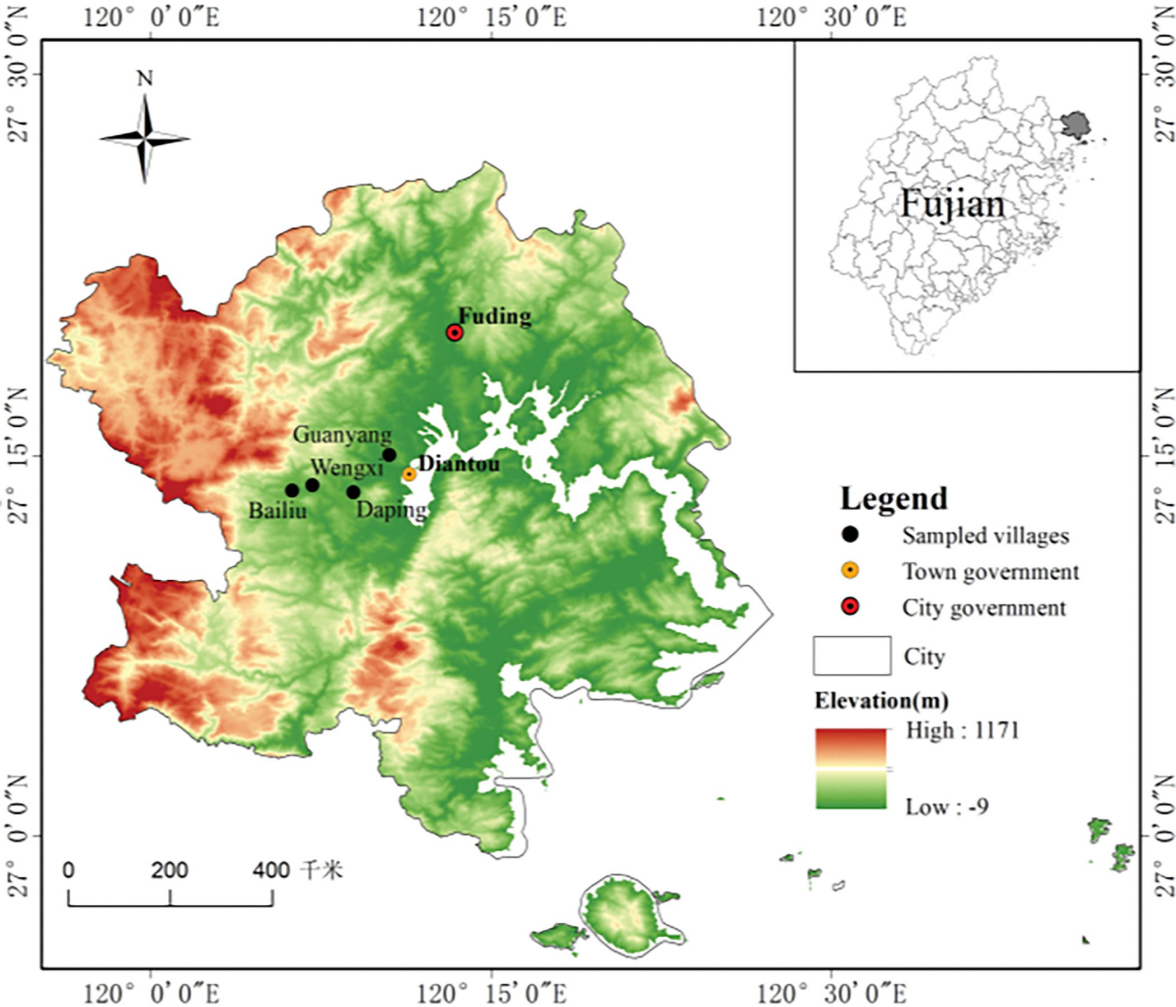
Location of study area. ***Note*:** The map is produced by the authors by referring to the blank base map derived from the internet (http://bzdt.ch.mnr.gov.cn/) owned by Map Technology Review Center, Department of Natural Resources of China and DEM data downloaded from the internet of “Geospatial Data Cloud”(internet address: http://www.gscloud.cn/).

Fuding City is a county-level city, which is located in northeastern *Fujian Province*, Southeast China (Fig 2). Three directions (i.e. northeast, northwest, southwest) are surrounded by mountains and the southeast faces the sea. It covers area of 1542.05 km^2^ and consists of 3 downtown sub-districts and 14 towns. In addition to the alluvial plains in the harbor area, the majority of area is mountains and hills, which account for 88.10% of the total area. The mountains include 8.95% of middle mountains and 25.46% of low mountains with elevations of 801–1649 m and 501–800 m, respectively. The acreage of hills and basins is relatively less, which has resulted in insufficient farmland. The farmland just accounted for 11.84% of the total area of land [31]. The farmland per capita was only 0.046 ha person^-1^ (including paddy field of 0.030 ha person^-1^), and 0.067 ha per agricultural labor in 2018. The scarcity of farmland forced rural households to develop mountainous resources to form many cash crop plantations. Fuding City has a subtropical marine monsoon climate with high mean precipitation of 1720 mm year^−1^ and warm annual temperatures (the annual average temperature being 19.2°C). The main natural vegetation types are *Pinus massoniana*, *Cunninghamia lanceolata*, *Cupressus funebris*, etc.

Tea farming has a long history in Fuding City by virtue of its suitable climate, soils and tradition. The acreage of tea plantations was 3,133 hectares in 1936. The prevalent application of agricultural technologies has propelled the remarkable sprawl of tea plantations since the 1970s. Then the tea plantation area increased to 5,238 hectares and 9,109 hectares in 1985 and 1995, respectively. With the expansion of tea plantations, it has been the leading industry of Fuding City. The yields and output value of tea reached 7,175 tons and 1,015 thousand USD in 1995. The total acreage of tea plantations was 15,104 hectares in 2020, which produced 32.40 thousand tons of tea, and 1.73 billion USD of output value in 2020. Tea plantations were mainly distributed in central and southwestern sections of Fuding. Three towns (including *Diantou*, *Bailin and Panxi*) are major tea-producing regions, which account for 46.34% of the total area. Among them, *Diantou Town* has the largest area of tea plantations, and its area accounted for 17.15% of the total in 2020.

## 3 Results

### 3.1 Proximate causes of landscape changes

#### 3.1.1 Tea plantation expansion

**Fig 3:**
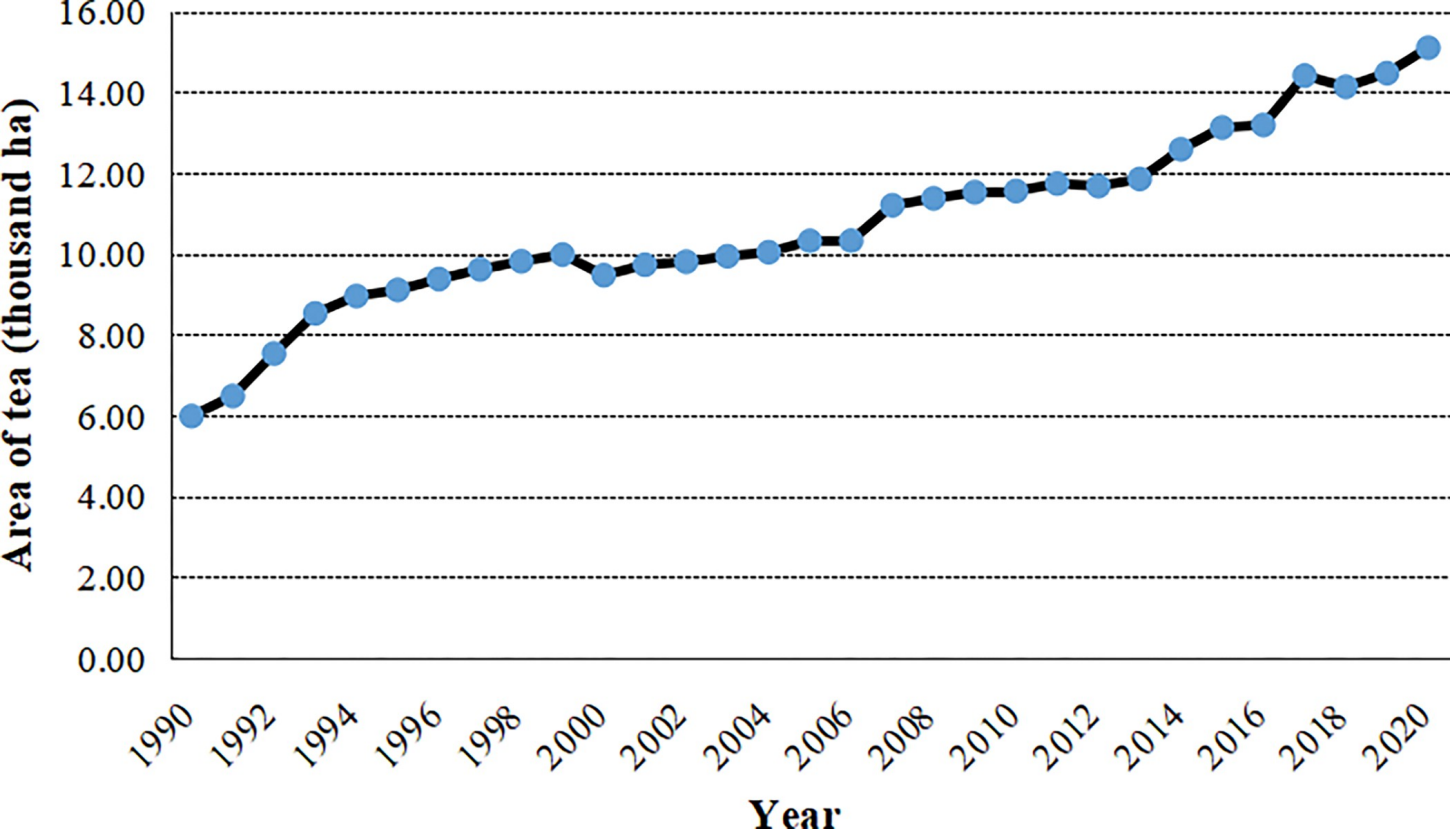
Changes in area of tea plantation during past four decades.

Tea plantations have experienced substantial expansion during past three decades. The total acreage of tea plantations increased from 5,237.60 ha in 1990 to 15,103.60 hectares in 2020, increasing by 1.88 times. The area of tea plantations accounted for 9.90% of the total area in 2020. There were two peak periods of tea expansion over the past four decades. The one was the rapid expansion beginning from 1991 to 1995, with the averagely annual newly planted tea area reaching 723.28 ha yr^**-1**^ (Fig 3). Another peak period of tea expansion was during 2014~2015, with annually incremental area of 635.90 hectares and annual expansion rate of 5.23%. Our investigation also indicated that 61.40% of surveyed rural households had experienced tea plantation expansion during the past ten years (Table 2).

**Table 2:** Manifestation of landscape changes driven by tea farming during 2010–2020.

Sampling village	Average tea PT (mu)	Tea PT expansion HH (%)	Paddy to tea nursery HH (%)	Paddy to tea PT HH (%)	Upland to tea PT HH (%)	Tea PT changed from abandonded* HH (%)
Guanyang	6.77	72.73	36.36 (19)	18.18 (10)	27.27 (14)	54.55 (25)
Wengxi	4.80	60.00	25.00 (10)	25.00 (18)	10.00 (5)	50.00 (32)
Daping	5.32	45.45	27.27 (4)	27.27 (15)	18.18 (4)	45.45 (20)
Bailiu	12.13	66.67	33.33 (18)	40.00 (20)	20.00 (10)	60.00 (80)
Average	6.60	61.40	29.82 (51)	28.07 (63)	17.54 (33)	52.63 (157)

***Note*:** area of tea plantations including acreage of tea nursery fields and common tea farming fields for producing tea leaves, the same below; parenthesis is the number of rural households; PT means plantations; HH means households; 1mu = 1/15 ha; * indicates the current intensively managed tea plantations changed from abandoned ones and extensively managed tea plantations inter-planting with fruit trees.

**Fig 4:**
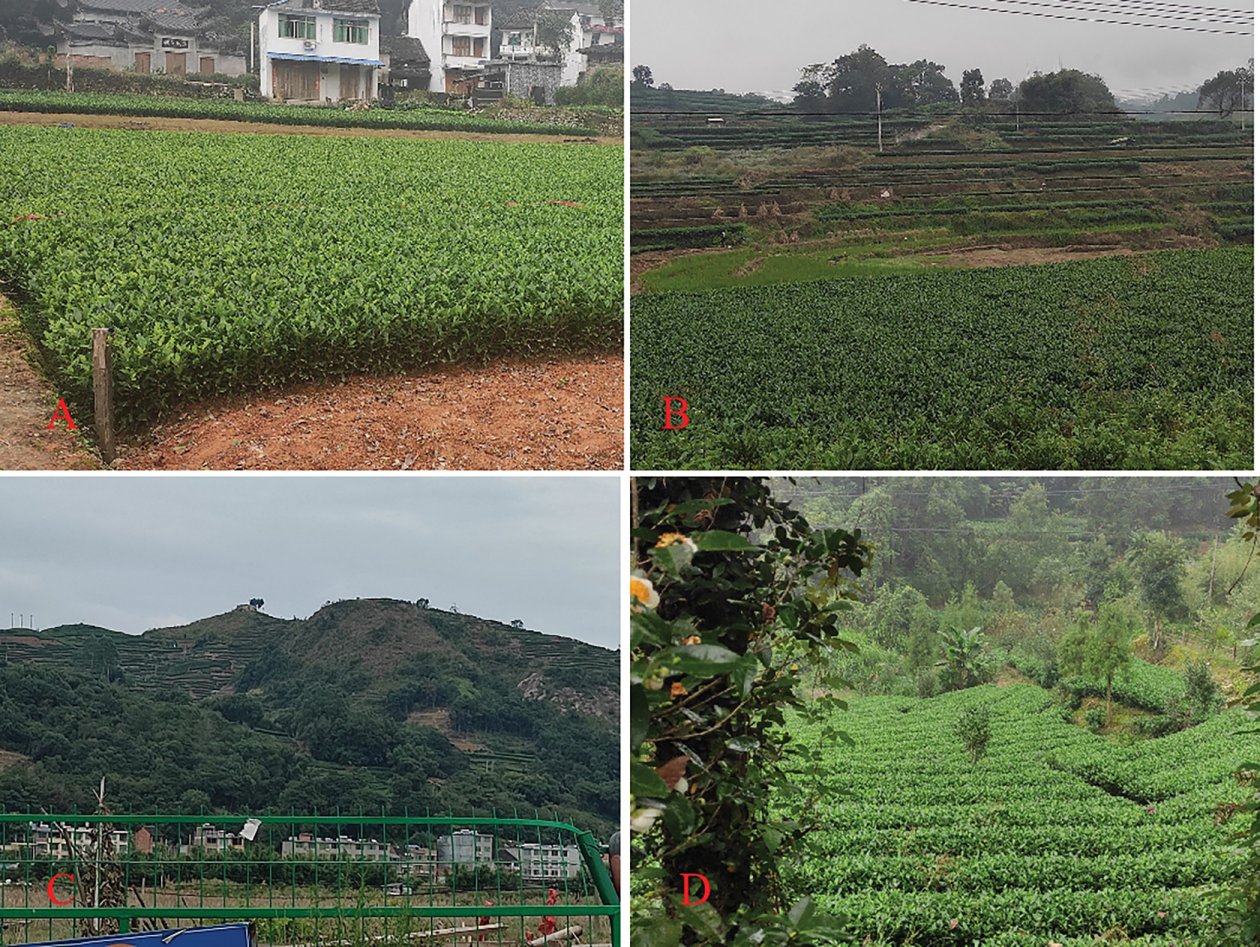
Landscape changes derived from four types of land use changes.

Our investigation showed that the expansion of smallholders’ tea plantations principally derived from three ways: paddy field to tea nursery field, paddy field to tea plantations, and upland (including woodland and bush wood land) to tea plantations (Fig 4). These land use changes have brought out rapid growth of the acreage of tea plantations in recent years. Results indicated that the area of newly increased tea plantations (including tea nursery field and common tea plantations) derived from above three kinds of land use changes accounted for 6.78%, 7.58%, and 4.39% in recent decade, respectively (Table 2). In other words, the percentage of tea plantation area changing from cropland and upland during the past decade was 14.36% and 4.39% respectively. The expansion rate of tea plantations was 18.74% in the past decade. And the transition rates of land use changes during the past decade were different in four survey villages. The transition rate could be calculated by the acreage of transition between tea plantations and other land types during 2010 to 2020 is divided by the original acreage of tea plantations in 2010.

#### 3.1.2 Intensification of tea plantations

Intensification of tea plantations has been the most important landscape changes in the past decade (Table 2). It generally means the higher input of tea plantations per hectare or higher tea production per hectare than traditional/previous management. The intensification of tea plantations has primarily been showed in two aspects: intensive management of abandoned tea plantations, and transition of tea-fruit inter-cropping to tea monoculture (Fig 4). Although the production of tea is few, due to the intensification of tea plantations which resulted in the number of tea plantations has been increasing so the landscape changes are dramatic. Our field survey proved that 39.61% of current smallholders’ intensively-farming tea plantations have been originally transformed from abandoned tea plantations or tea-fruit intercropping system during the past decade. Among the villages in survey, the transition rate of *Daping* (43.97%) is the most highest, and then is *Bailiu* (43.97%), *Guangyang* (33.5%), the *Daping’*s rate is the lowest (33.33%). Our in-depth interviews confirmed further our results that prevalent intensification of tea plantations was the most salient trait of landscape changes during the past decade.

### 3.2 Primary underlying drivers of landscape changes

**Fig 5:**
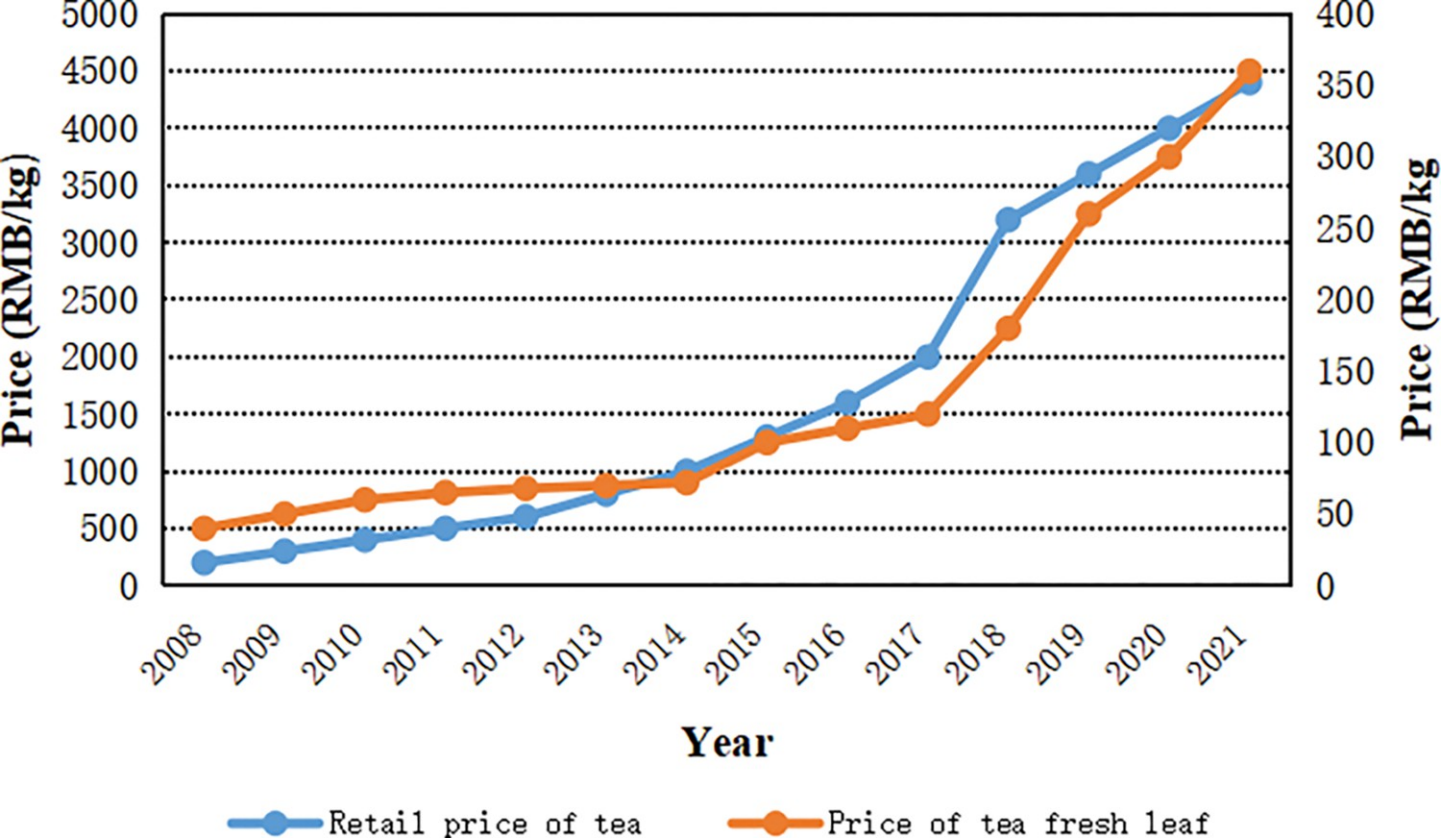
Trends of tea price in Fuding City, Southeast China.

**Fig 6:**
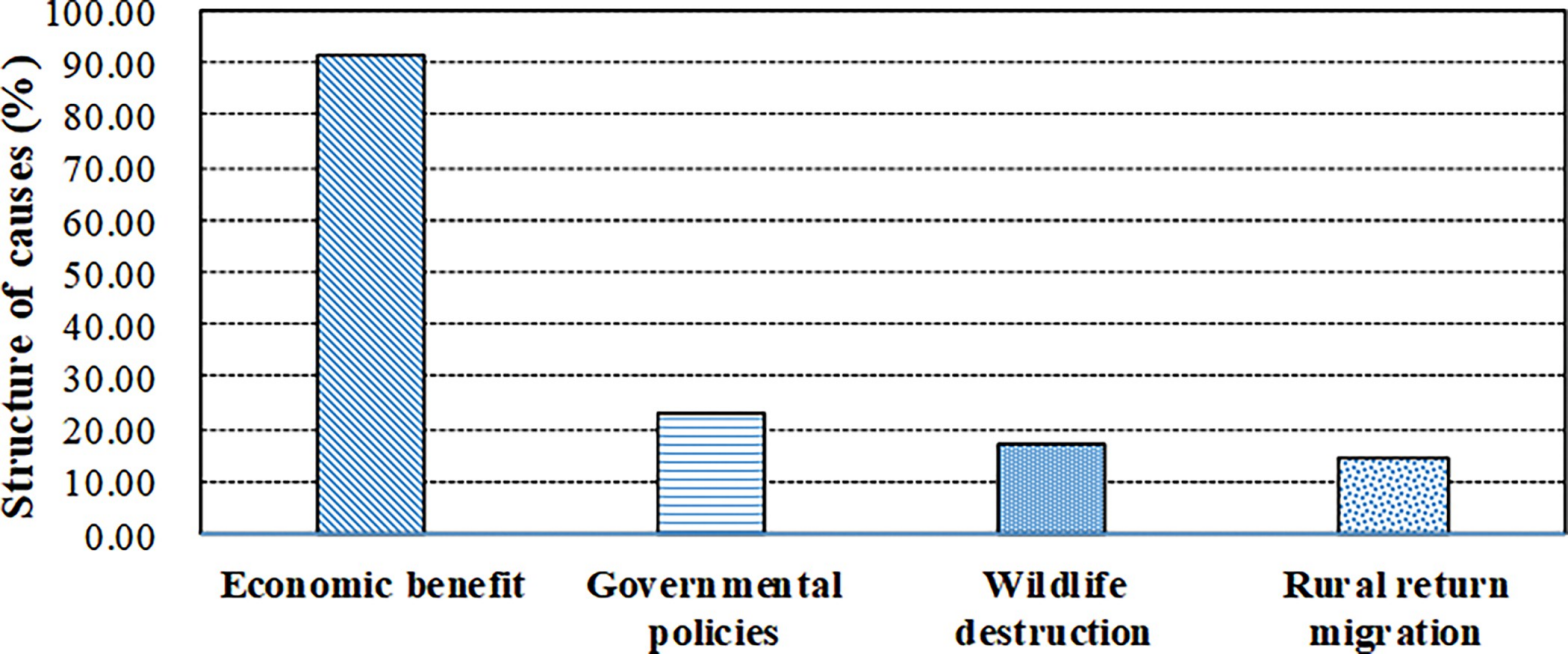
Primary causes of smallholders’ land use changes in tea plantation.

The expansion and intensification of tea plantations have been driven by the full interplay of economic, institutional, demographic and cultural variables. Our investigation has indicated that the increasing price and market demand of white tea was the most important reason for related land use changes. The market price of white tea has risen substantially during past ten years. The retail price of Pekoe Silver Needle tea with First Grade rose from 200 RMB kg_-1_ in 2008 (1 USD = 6.9451 RMB in 2008) to 4,400 RMB kg_-1_ in 2020 (1 USD = 6.8974 RMB in 2020, the same below), increasing by 21 times and with annual growth rate of 26.84%. High price of manufactured white tea which was produced in Fuding City has subsequently caused rapidly increasing price of fresh tea leaves. Correspondingly, the purchase price of tea tree’s topmost shoots increased from 40 RMB kg_-1_ in 2008 to 360 RMB kg_-1_ in 2020, increased by 8 times and with annual growth rate of 18.41% (Fig 5). Especially, the substantial appreciation of tea prices has triggered drastic land use changes in tea farms since 2015. The data of our questionnaires indicated that 91.43% of respondents regarded economic benefit of tea farming as one of primary causes of land use changes (Fig 6). While the rapid development of tea manufacture in local rural areas further promoted the expansion of tea plantation. For demographic factors, the severe aging of population in rural areas restricted the rural development by aggravating the labour scarcity for traditional crop farming. The tea plantation could provide an opportunity for rural elderly population to feed themselves. Compared to traditional crop farming, tea planting had relatively lower demands in physical power for agricultural labour. Our field survey showed that tea labours aged between 65~77 made up 22.22% of the total. Our survey showed that 14.29% of respondents considered returned migration as the principal cause of land use changes of tea plantation. As far as the institutional factors are concerned, the reform of property rights, especially the confirmation of long-term land contracting right for rural households, has greatly accelerated the initiative of farmers’ permanent investment in land, such as the transition of paddy field into tea plantation. In addition, the governmental promotion for tea planting, including providing subsidies for tea processing, and brand publicity, also facilitated the growth and intensification of tea plantation. And 22.86% of the respondents listed the policy and institutional factors as the main causes of land use change. Cultural factors are reported to underlie mainly economic and institutional forces in the form of attitudes and values of excessive focus on income growth and public unconcern towards the environment and sustainability. As for ecological factors, the ecological restoration and resultant wildlife proliferation brought about substantial impacts on crops. Transition from grain crops to tea tree is a coping strategy to reduce the wildlife damages. Our survey proved that 17.14% of respondents considered destruction of wild boars as the main cause of land use changes (Fig 6).

### 3.3 Livelihood outcomes of landscape changes

#### 3.3.1 Employment and incomes

Monoculture tea is considered as the most profitable land use activity in the study area; because it’s economic returns are higher than other land use systems (e.g., orchards, grain cropping, and forests). Tea growing and processing is the leading industry of Fuding City, which creates substantial job opportunities. There are averagely about two self-employed labours in each smallholder who are engaged in tea farming. The middle-aged and elderly labours, which have less employment opportunities in non-farm sectors of urban areas, are the principal tea farming labours. The average age of self-employed tea farming labours is 55.21 years old; the difference among four sampling villages is little. The tea farming labours whose age is between 45~65 make up 61.5% of total self-employed labours, age between 60~ 77 accounts for 41.88%, age between 65~77 make up 22.22% (Table 3). Moreover, the thriving tea industry produced the market of casual labourers. Smallholders with large-scale tea plantations or less workforces tend to employ some temporary workers to pick up tea leaves in busy Spring. Many local laborers with less tea plantations or laborers from adjacent counties are hired to perform temporary work in tea farms. The average number of employed casual laborers for each smallholder in *Guangyang*, *Wengxi*, *Daping*, *and Bailiu* is 2.2, 0.37, 1.27, and 2.60, respectively (Table 3). In a word, tea became the most important cash crop and tea farming was the leading employment sector in study area.

**Table 3:** Employment and incomes deriving from tea farming in Fuding City.

Sampling village	Self-employed tea labour (pcs)	Average age of labour(y/o)	Tea income (thousand RMB household^-1^)	Tea income to total (%)	Hired casual labourer	labour cost (thousand RMB household^-1^)
Guanyang	2.00	56.3	66.00(41.13)	81.49	2.20	11.20
Wengxi	2.20	56.02	53.50 (23.78)	67.44	0.37	2.20
Daping	1.91	53.05	46.41 (21.74)	64.05	1.27	6.90
Bailiu	2.00	55.47	76.15 (43.82)	86.73	2.60	15.30
Average	2.05	55.21	60.50 (37.36)	74.57	1.39	7.60

***Note***: parenthesis is the value of standard deviation; tea income including incomes from selling tea leaves, and tea seedlings; labour cost means costs of hiring casual labourer in tea plantation.

Incomes from tea plantations constitute the leading parts of smallholders’ revenue. The self-employed laborers could gain 60.50 thousand RMB household^-1^ (1 USD = 6.4515 RMB in 2021) which constitutes of approximately 75% of total household income (Table 3). There are some significant differences in smallholders’ tea earnings among four sampling villages because of the discrepant natural endowments. Labor costs of hiring casual laborers are the primary expense for picking up tea leaves. The labour costs of tea plantations in *Guangyang*, *Wengxi*, *Daping*, *and Bailiu* are 11.20 thousand RMB household^-1^, 2.20 thousand RMB household^-1^, 6.90 thousand RMB household^-1^, and 1.53 thousand RMB household^-1^, respectively (Table 3). The differences of cost among four sampled villages could be attributed to the scale of tea farms and scarcity of family labours. Our in-depth interviews showed that the overall tea-related revenue, including tea farming, tea processing, and tea marketing, accounted for more than 90% of total rural incomes.

More importantly, tea farming has provided a viable livelihood option for the disadvantaged groups, including the elderly (esp. more than 65 years old), the slightly handicapped, the chronic patients, the unemployed person (esp. returning from urban areas to hometown), and the rural unmarried middle-aged and aged men, because management of tea plantations do not need professional skills and high-intensity work (Fig 1). That is to say, tea farming has lower labour requirements than alternative crops. Especially for those poor mountains with limited flat cropland, the significance of growing tea is fundamental for the livelihoods of rural disadvantaged people.

#### 3.3.2 Improvement in living standards

Living standards of smallholders who own tea plantations have been improved owing to comparatively high incomes (Fig 1). Daily expenses can be used as a index to represent the level of living standards. Our study indicated that the annually average living costs of rural permanent residents were 8,508 RMB person^-1^ yr^-1^, which was massively higher than other rural areas without tea plantations (Table 4). Moreover, the incomes from tea plantations could nearly cover all the basic living costs of smallholders. Except for four smallholders, 96.49% of respondents reported that their tea earnings could cover all the household rural living costs. The percentage of smallholders’ average living costs to total tea incomes was 31.41%, 35.57%, 40.90%, and 40.87% for *Guangyang*, *Wengxi*, *Daping*, *and Bailiu* village, respectively (Table 4). The majority of rural households mainly acquired food (such as grain, and vegetables) through market, because most cropland was changed into tea plantations.

**Table 4:** Living standards and consolidation of livelihood assets of sampled rural households.

Sampling village	Average living cost (thousand RMB person^-1^ yr^-1^)	Living cost (thousand RMB HH^-1^ yr^-1^)	Expenses to total tea income (%)	Housing area (m^-2^ person^-1^)	Owning houses in townships or cities (%)
Guanyang	7,860	20.73	31.41	46	54.55
Wengxi	7,584	20.1	37.57	45	45.00
Daping	8,352	18.98	40.90	42	72.73
Bailiu	10,320	30.96	40.87	50	80.00
Average	8,508	22.86	37.87	46	61.40

***Note***: living cost mainly including costs of daily necessities such as food, clothing, energy, tap water, and other necessary subsistence spending; HH means household; 1 USD = 6.4515 RMB in 2021; housing area means the housing area per capita of rural permanent residents; owning houses means the surveyed householders or their children possessing the property rights of houses in local townships or cities.

Our investigation demonstrated that great livelihood improvement has taken place since the recent decade. We found that more than 90% of respondents’ livelihood independence, which was measured by the question “if your living costs could be mainly covered by tea incomes? (yes = 1, no = 0)”, was substantially promoted due to the tea management (Fig 6). Moreover, consumption costs in food and real state rose tremendously, which revealed the livelihood improvement in different dimensions. Questionnaires indicated that 36.36%, 55.00%, 36.36%, and 40.00% of respondents in *Guangyang*, *Wengxi*, *Daping*, *and Bailiu* village respectively reported the remarkable increase was in food spending. Food spending is measured by the cost of food gained through directly purchasing from the market and the converted value of self-grown grain and livestock for self-consumption. And 45.45%, 60.00%, 54.54% and 66.67% of surveyed smallholders in *Guangyang*, *Wengxi*, *Daping*, *and Bailiu* village respectively have derived substantial growth in housing expenses (including buying or building new houses, renovating, and decoration) from tea farming (Table 4). The average percentage of rural households owing houses in townships and cities had increased from about 10% to 61.4% during the past ten years (Table 4).

#### 3.3.3 Upgrade in livelihood assets and livelihood sustainability

**Fig 7:**
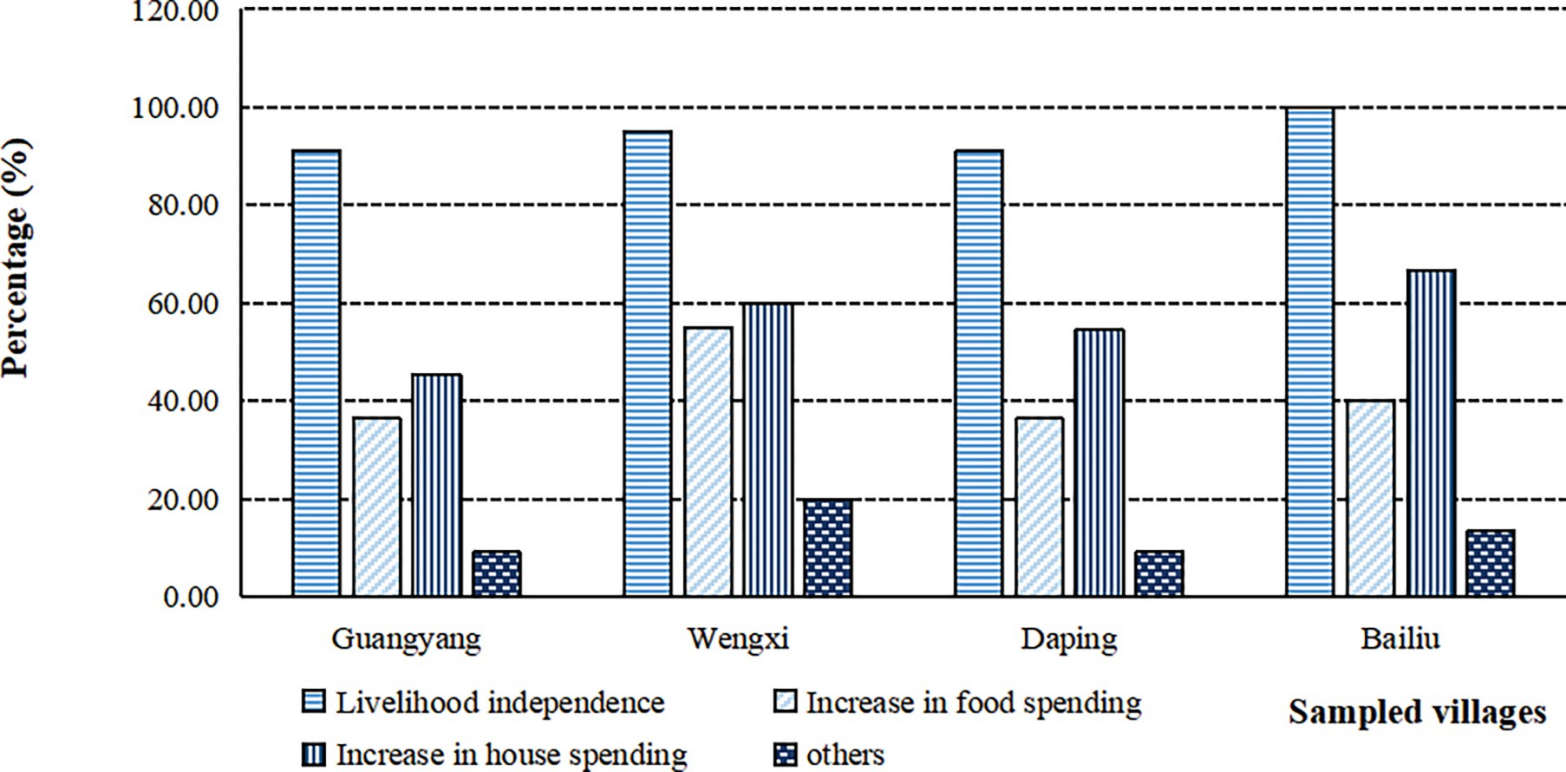
Livelihood improvement attributed to tea plantation.

Stable and high economic benefits derived from tea plantations have promoted the upgrade of rural households’ livelihood assets, especially the houses, which in turn propelled the livelihood sustainability (Figs 1 and 7). It is a Chinese long-standing tradition that parents need to build or buy a new house for their immature sons before they establishing a new family. While the location and expenditure of the new house depends on the family’s financial situations. Our investigation showed that the cost of building a 4-storery townhouse in rural village and township is about 200~300 thousand RMB, and 600~700 thousand RMB respectively. The expense of buying a 120 m_2_ apartment in Fuding City is about 1000 thousand RMB. Our survey demonstrated that the percentage of owning houses in townships or cities in *Guangyang*, *Wengxi*, *Daping*, *and Bailiu* village was 54.44%, 45.00%, 72.73% and 80.00%, respectively (Table 4). The house location and value is strongly associated with the provision of public service, such as education, medical service and social security. Thus, the offspring’s settlement in townships and cities other than rural villages has implied the promotion of livelihood sustainability. In addition, human assets, which includes knowledge, skills, entrepreneurship of tea plantations, processing and marketing, have been tremendously enhanced by transmission from ancestors, training, and learning by doing.

## 4 Discussion and conclusions

### 4.1 Landscape changes driven by tea plantations

The results confirmed that great landscape changes had taken place in the study area during the past decades. The landscape changes were not only manifested in tea plantation expansion, but also in intensification of tea plantations. Firstly, the expansion of tea plantation has experienced two upsurges since the 1990S. There were about 20% of tea plantations have been expanded in recent ten years. The tea plantation expansion has been manifested in three types of land use changes, including paddy field to tea nursery, upland to tea plantations and paddy field to tea plantations during past ten years. These results were consistent with previous studies that new monoculture tea plantations are mainly established in places previously used as woodland [5, 7, 17] (Xiao et al., 2015; Su et al., 2016; Su et al., 2017), and tea nursery plantations are primarily observed in prior cropland [7, 32].

Secondly, the intensification of tea plantations has been a more prevalent pattern of landscape changes in recent decade. About 40% of existing tea plantations have been transformed from abandoned ones or extensively managed tea plantations (such as inter-planting with other crops) during past decade. Agricultural intensification is also a land cover modification process in which the changes of land use intensity alter the land cover without necessarily changing its land use. Field surveys, such as questionnaire surveys and site investigation, are generally utilized, whereas the method of RS and GIS are often not appropriate to identify some of these land cover changes in mountainous areas with highly fragmented terrains. High-resolution of remote sensing imagery and appropriate research methods may overcome these research barriers in future. The results were consistent with the past studies that agricultural intensification had caused widespread landscape changes by substituting original heterogeneous and diverse landscapes for homogeneous monoculture landscapes [33, 34]. The results were also similar to previous research that land expansion generates remarkable agricultural landscape changes in European countries [35, 36]. However, the landscape changes driven by intensification of tea plantations have been less discussed.

### 4.2 Driving forces of landscape changes

Two proximate causes of landscape changes were identified: tea plantation expansion, and intensification of tea plantations, following the concept of Geist and Lambin [30]. Proximate causes are human activities or immediate actions at the local level that directly affect agricultural landscapes. Underlying driving forces of landscape changes are fundamental social processes, which underpin or drive the proximate causes [30, 37, 38]. Economic benefit, governmental policies, wildlife destruction on grain crops and rural return migration, are important causes of expansion and intensification of tea plantations. These results were in accordance with previous studies that economic incentive and governmental policies are significant determinants of cash crop expansion [4, 7, 17]. The results were also identical to prior researches that substituting grain crops for unpalatable cash crops (such as chilli) can reduce human-wildlife conflicts [39, 40]. Our research also verified that cash crop farming provides a feasible livelihood strategy for aged return laborers [41].

### 4.3 Livelihood impacts of landscape changes

Our study indicated that the livelihood impacts of expansion and intensification of tea plantations were substantial, including increasing employment and incomes, raising living standards, and enhancing livelihood assets at present. Especially, the expansion of tea plantations was significant in promoting living standards of the rural aged, who had less viable livelihood options. Persistent cash earnings are the principal livelihood benefit of outspread tea plantations. These results agreed with previous studies which widely affirmed that cash crop farming can alleviate rural poverty through increasing household earnings [17, 27, 42, 43]. Economic benefits are generally recognized as the most prominent livelihood benefit of cash crop expansion. Smallholders’ living standards and food security can also be enhanced through more incomes derives from cash crop management [24, 44]. Improvement of livelihood assets, including physical assets (esp. cash crop plantations, facilities for production, furniture appliances), human assets (such as knowledge, skills, health) and social assets (such as social networks and community trust), can be also achieved through the expansion of cash crops [24].

The livelihood sustainability has been highlighted that the livelihood sustainability of smallholders’ off-springs has been promoted by upgrading public services, especially better education. This result is similar with previous research that cash crop farming can promote smallholders’ livelihood sustainability through supporting children’s more education and cultivating well-qualified primary school teachers [44]. For the majority of populous Asian developing countries, such as China, Malaysia, and Indonesia, the smallholders’ livelihood vulnerability is high owing to the small farm scale, low productivity and fluctuated market price of farm products. The children’s livelihoods upgrading from small-scale agriculture to urban knowledge-based non-farm industries can bring out great improvement in livelihood sustainability and social status [44, 45]. On the other hand, the transition from diversified agriculture to mono-tea farming may impair farmers’ livelihood resilience. Given the abrupt outbreak of COVID-19 epidemic and long lockdown, it should be the alert that the livelihood resilience of smallholders needs to be improved, because excessive land use/cover turns into tea plantations will leads to excessive dependence on market to obtain daily food, including cereal, vegetables, poultry, etc. Moreover, the research period is coincidence with the stage of high market price and increasing demands of white tea in China. The livelihood situations may be quite different if they encounter weak tea market demands and low tea price.

Landscape changes’ impacts on poverty alleviation, especially for the aged rural population, were highlighted in our study. Results of our study demonstrated that most aged farmers gained a decent life through the new lucrative livelihood strategies. Participation in the cash crop farming is the premise to benefit the poor. Our investigation showed that nearly all rural households had tea plantations because of the equal land allocation in the 1980s, less investment, and low labor requirements in skills and physical activities. These results were inconsistent with previous studies, which found that the poorest families maintained most paddy fields and did not converted their land into rubber or oil palm plantations [44]. The poorest rural households are usually excluded from developing cash crop plantations. Non-farm households in rural areas of Indonesia have to work in cash crop plantations to improve their livelihoods [46]. Different land allocation policies and labour requirements may be the main reasons of the research differences. Especially, the aged laborers were less employed in oil palm and rubber farms owing to high requirements from the employers.

### 4.4 Policy implications

There are some policy implications in our study. Firstly, the supportive policies of land transfer and concentration, which are national policies issued by the central government of China should be acted flexibly according to local conditions. Our study corroborated the significance of tea farming for the aged rural population. The one fits all policies but without considering local farmers’ livelihoods needs to be avoided. According to the National Bureau of Statistics and the Third Chinese National Land Survey, cropland per agricultural labour was only 0.72 ha, and the cash crop plantations per agricultural labour was just 0.11 ha in 2020. It is essential to promote land concentration and agricultural intensification in order to increase farming productivity in flat plain areas, such as Northeast China Region, Central China Region, and Northwest China Region. But it is not applicable to extend radically in steeply mountainous regions with relatively low urbanization rate.

Secondly, it is necessary to promote food self-sufficiency to a certain extent by implementing some restrictive and incentive policies. Livelihood resilience has been significantly impaired by an overwhelming expansion of tea plantations. Rural households are highly dependent on market to acquire daily foods, such as grain, vegetables, and poultry meat. Global outbreak of pandemic, Russia invasion in Ukraine and induced grain supply chain disruptions have warned that food security on different scales should be reinforced. The entire dependence on external market to guarantee food supply in rural areas is not reasonable.

Last but not least, restrictions on tea plantation expansion and financial subsidies for reclamation of low-quality tea plantations can be adopted to rectify the excessive land use transition. These remedial measures can also partly realize trade-off between economic benefit and ecological cost.

## Supporting information

S1 Data Survey data. (XLSX)
